# Estimating direct effects of parental occupation on Spaniards’ health by birth cohort

**DOI:** 10.1186/s12889-016-3997-6

**Published:** 2017-01-05

**Authors:** Jaime Pinilla, Beatriz G. Lopez-Valcarcel, Rosa M. Urbanos-Garrido

**Affiliations:** 1 Department of Quantitative Methods for Economics and Management, University of Las Palmas de Gran Canaria, Las Palmas de Gran Canaria, Spain; 2 Department of Public Finance, School of Economics, Complutense University of Madrid, Campus de Somosaguas s/n, 28223 Pozuelo de Alarcón, Spain

**Keywords:** Intergenerational transmission, Self-assessed health, Socioeconomic status, Cohort effects

## Abstract

**Background:**

Social health inequalities in adult population are partly due to socioeconomic circumstances in childhood. A better understanding of how those circumstances affect health during adulthood may improve the opportunities for reducing health disparities. The objective of this study is to investigate the effect of parental socioeconomic status, which is proxied by occupation, on adult Spaniards’ health by birth cohort. The analysis will allow checking not only the direct impact of parental occupation on their offspring’s health, but also whether inherited inequality has been reduced over time.

**Methods:**

We use data from the Bank of Spain’s Survey of Household Finances on Spanish households from 2002 to 2008. Sequential models were used to estimate the influence of the father’s and mother’s occupation on their offspring’s health, trying to disentangle direct from indirect effects. With a sample of 26,832 persons we consider effects for four different cohorts by birth periods ranging from 1916 to 1981.

**Results:**

The results show that parental occupation has a significant direct impact on individuals’ health (*p* < 0.01). The effect of father’s occupation exceeds that of mother’s. For those born before 1936, the probability of reporting a good health status ranges from 0.31 (95% confidence interval (CI) 0.14–0.48), when fathers were classified as unskilled elementary workers, to 0.98 (95% CI 0.98–0.99) when they were managers or mid-level professionals. For those born during the period 1959–1975, those probabilities are 0.49 (95% CI 0.39–0.59) and 0.97 (95% CI 0.96–0.98), respectively. Therefore, health inequalities linked to parental socioeconomic status have been noticeably reduced, although discrimination against unskilled workers persists over time.

**Conclusions:**

Great progress has been made in the health area during the twentieth century, so that the impact of parental socioeconomic status on individuals’ health has been significantly tempered for those at the bottom of the social scale. However, more efforts focused on the improvement of living conditions for most socioeconomically disadvantaged are needed in order to further reduce social inequalities in health.

## Background

It has been well established that social health inequalities in adult population are partly due to socioeconomic circumstances in childhood [1–22]. Two basic hypotheses explain the relationship between social status in early life and adults’ health. According to the life course hypothesis, social background directly influences health in adulthood since those with inferior living conditions during childhood and adolescence are more likely to experience poor health later in life [2, 4–6, 16, 20, 23]. Moreover, the pathway hypothesis suggests that parental socioeconomic circumstances indirecty influence health in adulthood through the transmission of socioeconomic status (SES) and the investment in children’s human capital [7, 8, 12, 17, 22].

The evidence of how parental SES affect Spaniards’ health is scarce. Tubeuf and Jusot [11] and Bricard et al. [18] show how parents’ social status and initial conditions in childhood influence adults’ health in Spain and some other European countries, although they do not distinguish between direct and indirect effects. They also find that the contribution of family background to social health inequality in Spain is more important than the contribution of individual status. Flores and Kalwij [21] also prove for a group of European countries including Spain that favourable early life circumstances are associated with a higher level of education and also with better health in adulthood, even when education is controlled for, thus showing the existence of both direct and indirect effects of parental status on health. These previous studies use data from the Survey of Health, Aging, and Retirement in Europe (SHARE), which only includes adults in pre-retirement age. This paper uses a different database, the Spanish Survey of Household Finances (EFF by its Spanish acronym), which represents the entire adult population of Spaniards. Also, this dataset allows checking if effects of parental SES on individuals’ health significantly differ by cohort. To the best of our knowledge, this issue has not been previously addressed.

The objective of this study is to investigate the effect of parental socioeconomic status, which is proxied by occupation, on adult Spaniards’ health by birth cohort. The analysis will allow checking not only the direct impact of parental occupation on their offspring’s health, but also whether inherited inequality has been reduced over time. A better understanding of how social circumstances in childhood affect health during adulthood may improve the opportunities for reducing health disparities. Furthermore, the research on whether the transmission of social inequalities has been tempered across the twentieth century will help us to determine the redistributive efficacy of Spanish public policies over time.

## Methods

### Dataset and sample definition

The EFF is a survey undertaken by the Bank of Spain that is included in the Spanish National Statistics Plan. There have been four waves: the first started in 2002 (data collected in 2002 and 2003), the second in 2005 (2005–2006), the third in 2008 (2008–2009) and the most recent in 2011. Each wave surveyed about 5500 households, representing approximately 15,000 people, which accounts for a representative sample of the Spanish population. In our analysis, we pooled the data from EFF2002, EFF2005 and EFF2008. Previously, we performed tests of homogeneity to check for wave effects. We excluded data from 2011, as the effects of the economic crisis on income and wealth, as well as on health measures, might bias the results of the study. This database is quite rich in economic information about families, including those with a privileged socioeconomic status.

The EFF questionnaire mainly provides economic information about income, net wealth and consumption at the household level, but it also includes individual socio-demographic characteristics such as age, sex, educational level, occupational category, self-assessed health (SAH) and past circumstances associated with parents’ social status for the household reference person and for his/her partner. In particular, the EFF includes information about occupational category that parents have (or had for most of their working lives). This data thus allows us to link individuals’ SAH, not only with their current socioeconomic situation, but also with their socioeconomic background, since parental occupation may be a good proxy of the childhood socioeconomic group.

Our sample was limited to the household reference person (usually the person in the household who handles financial issues) and his/her partner, because in the questionnaire only these individuals are asked for information about their parents. We have truncated the sample to the age interval 28–86 at the time of the survey. People younger than 28 have been excluded after we developed a joinpoint analysis [24] of the rates of people reporting bad health at each age. Also, individuals aged over 86 have been ignored since they show a high variability in their self-assessed health.

### Empirical model specification and estimation method

We are interested in quantifying the direct impact on adults’ health of childhood circumstances and social background, given by their parents’ occupation. However, as we mentioned before the relationship between parental situation and an individual’s health may take place through indirect channels, as parents’ SES may influence offspring’s educational level and occupation. Therefore, the econometric method we adopt to estimate the direct effects of parental occupation on health controls for indirect effects via education and occupation. Following Trannoy et al. [10], we successively estimated three discrete choice equations, where dependent variables are educational level, occupational category and health, respectively. From the first two discrete choice equations we calculate the ‘generalized residuals’ (*U*_*s*_), which are used as proxies for unobservable variables in the respective next equations. The final model is then represented by Eqs. (1) to (3).

1 $$ \fontfamily{Arial Narrow}{Education}={\fontfamily{Arial Narrow}{f}}_{\fontfamily{Arial Narrow}{1}}\left(\fontfamily{Arial Narrow}{D},\ \fontfamily{Arial Narrow}{C},\ \fontfamily{Arial Narrow}{T},\ \fontfamily{Arial Narrow}{C}*\fontfamily{Arial Narrow}{T},\ {\fontfamily{Arial Narrow}{u}}_{\fontfamily{Arial Narrow}{1}}\right) $$

2 $$ \fontfamily{Arial Narrow}{Occupation}={\fontfamily{Arial Narrow}{f}}_{\fontfamily{Arial Narrow}{2}}\left(\fontfamily{Arial Narrow}{D},\ \fontfamily{Arial Narrow}{C},\ \fontfamily{Arial Narrow}{T},\ \fontfamily{Arial Narrow}{C}*\fontfamily{Arial Narrow}{T},\ \widehat{u_1},{\fontfamily{Arial Narrow}{u}}_{\fontfamily{Arial Narrow}{2}}\right) $$

3 $$ \fontfamily{Arial Narrow}{Health}={\fontfamily{Arial Narrow}{f}}_{\fontfamily{Arial Narrow}{3}}\ \left(\fontfamily{Arial Narrow}{D},\ \fontfamily{Arial Narrow}{E},\ \fontfamily{Arial Narrow}{C},\ \fontfamily{Arial Narrow}{T},\ \fontfamily{Arial Narrow}{C}*\fontfamily{Arial Narrow}{T},\ \widehat{u_1},\widehat{u_2},{\fontfamily{Arial Narrow}{u}}_{\fontfamily{Arial Narrow}{3}}\right) $$

where D represents the vector of demographic characteristics, C the childhood circumstances, T the time variables (birth cohort), and E the individual economic status. According to Eq. (1), educational attainment depends, besides demographic characteristics, on childhood circumstances (that will be proxied by father’s and mother’s occupation) and time variables. Since one of our aims is to analyze if the dynastic effect varies over time, we also include in all the equations cohort dummies and interaction variables between parental occupation and cohorts. In Eq. (2), personal occupational category is again regressed on demographic variables and parents’ occupation legacy, besides on cohort variables. We also include the generalized residual of Eq. (1). The term $\widehat{u_1}$ indicate the portion of personal educational attainment which is not attributable to parental circumstances or demographic factors. Finally, Eq. (3) specifies that health depends, besides demographic characteristics, on the current economic situation of the household (E), on the chilhood circumstances and cohort variables as well as on education and occupation, which have been previously purged of parental influence through generalized residuals ($\widehat{u_1}$ and $\widehat{u_2}$). Thus, the coefficients of parental variables in Eq. (3) attempt to measure the direct effect of the father’s and mother’s occupation on the descendant’s health.

As the interaction effects are conditional on the values of the predictor variable, even if we observe a significant coefficient the effect may be statistically insignificant across all values of the regressor [25]. Thus, we calculated the adjusted probabilities for interaction terms in Eqs. (1)-(3). The probabilities have been evaluated for the observed values of the sample individuals, and then we calculated the average (Average Partial Effects, APE). We also estimated the 95% CI for each probability by delta method. In the estimation process we have incorporated the weight of each individual according to the sample design.

Equation (1) is estimated as an ordered probit model since, as we explain in the next subsection, we define four categories for educational level. Moreover, Eqs. (2) and (3) are estimated as binary probits. Due to the impossibility of obtaining a non-arbitrary ranking of social status [10], we only distinguish in Eq. (2) high from low occupational status. Finally, we used the approximate likelihood test of equality of coefficients across response categories to test the appropriateness of using a binary dependent variable in Eq. (3), instead of an ordinal variable. Since the non-linear specification does not allow direct estimation of the residuals $\widehat{u_1}$ and $\widehat{u_2}$, we calculated the generalized residuals, which correspond to the conditional expected value of residuals given the outcome [26].

### Definition of variables

Dependent variables in Eqs. (1) to (3) have been defined as follows. Firstly, educational level has been categorized into four groups: ‘illiterate and primary education’, ‘lower secondary schooling’, ‘higher secondary schooling’ and ‘specialised vocational training and university degree’. The occupational category has been defined as a dummy variable taking a value of 1 for high occupational status (top managers and mid-level professionals, ISCO 1-4), and 0 otherwise. Finally, SAH is used as a proxy for health stock, which was assessed through a single question: ‘How is your health in general?’, with five possible answers: very good, good, acceptable, poor and very poor. These five categories have been reclassified in two by defining a dummy variable taking value of 1 for good or very good health, and 0 for fair, poor or very poor health. We will refer to these categories as ‘good’ and ‘bad’ health throughout the text.

The vector D of demographic characteristics includes age and sex. Childhood circumstances (C) have been proxied by parental occupation, since it is the only variable included in the EFF about the social background of individuals during their early lifes. Occupations are described with the one-digit ISCO (International Standard Classification of Occupations), which distinguishes ten main groups of occupations, plus one for homemakers.

Fathers’ occupations are classified into five groups: (1) ‘top level managers in the public or private sector, technicians and professionals, intellectuals or scientists’ (ISCO 1 + 2); (2) ‘supporting technicians and professionals, administrative staff’ (ISCO 3 + 4); (3) ‘armed forces’ (ISCO 10); (4) ‘skilled workers in service, agriculture, fishing, manufacturing, construction or mining industries’ (ISCO 5-8); and (5) ‘unskilled elementary workers’ (ISCO 9). As there are not any homemaker fathers in the sample, that category is not defined. Due to low frequencies in some occupational categories, we distinguished only three categories for mothers in our final models: (1) top and middle-level professionals (ISCO 1-4); (2) other workers, including skilled and elementary occupations (ISCO 5-10); and (3) homemakers.

Individuals in the sample, aged 28–86 at the time of the survey, were born from 1916 to 1981. We have defined our T variable by distinguishing four cohort categories related to the Spanish political and economic context: 1) pre-war cohort, before 1936; 2) war and post-war cohort, born in 1937–1958; 3) Stabilization Plan -Franco’s 1959 economic reform plan- cohort, born in 1959–1975; and 4) democracy cohort, born after 1975 (since Franco died in November 1975).

Finally, the current economic situation of individuals (E) is measured by two variables: family income and family wealth. We calculated quartiles of household equivalent income and wealth by using the OECD equivalence scale (which assigns a value 1 to the household head, 0.7 to each additional adult and 0.5 to each child). The income variable is obtained as the sum of labour and non-labour incomes for all members of the household. The net wealth variable is calculated from the value of a household’s real assets: main residence, other real estate properties, jewelry, works of arts, antiques, and the value of businesses related to self-employment. The first quartile also includes those households with negative wealth (net debt).

## Results

Table 1 shows the descriptive statistics of the estimation sample (frequency distributions for discrete variables, mean and standard deviation for continuous ones and for income and wealth quartiles). Standard error estimates are produced using Taylor linearization. The number of individuals in the sample who are the household’s reference person or his/her partner is 28,980. After removing observations with missing data, our estimation sample has 26,832 individuals aged 28–86. All the calculations have been made by using the sampling weights.

**Table 1:** Definition of variables and descriptive statistics

Variables	Recoded variables	Definition	Mean^a^ (*n* = 26,832)
Dependent variables			
Educational level	Education^b^	1 if illiterate or primary education, 2 if lower secondary schooling, 3 if higher secondary schooling, 4 if specialized vocational training or university degree	(1) 40.02% (2) 18.42% (3) 15.34% (4) 26.22%
Occupational status	Top_workers	1 if manager, mid-level professional or supporting worker, 0 otherwise	14.20%
Self-assessed health	SAH	1 if very good or good health is reported, 0 otherwise	79.50%
Independent variables			
Age	Age	Age in years	45.99 (14.52)
Sex	Female	1 if female, 0 otherwise	52.44%
Equivalent household income^c^	Income_Q1 (omitted category)	1 if the person belongs to the 1^st^ income quartile, 0 otherwise	5460.38 (40.80)
	Income_Q2	1 if the person belongs to the 2^nd^ income quartile, 0 otherwise	10284.62 (40.76)
	Income_Q3	1 if the person belongs to the 3^rd^ income quartile, 0 otherwise	16746.49 (72.10)
	Income_Q4	1 if the person belongs to the 4^th^ income quartile, 0 otherwise	37524.9 (693.05)
Equivalent net household wealth^c^	Wealth_Q1 (omitted category)	1 if the person belongs to the 1^st^ wealth quartile, 0 otherwise	19014.56 (492.31)
	Wealth_Q2	1 if the person belongs to the 2^nd^ wealth quartile, 0 otherwise	77213.64 (629.88)
	Wealth_Q3	1 if the person belongs to the 3^rd^ wealth quartile, 0 otherwise	167070.5 (1755.69)
	Wealth_Q4	1 if the person belongs to the 4^th^ wealth quartile, 0 otherwise	540931.7 (21606.4)
Father occupation	F_Manager (omitted category)	1 if manager or mid-level professional (ISCO 1 + 2), 0 otherwise	10.22%
	F_Supporting	1 if supporting worker (ISCO 3 + 4), 0 otherwise	15.70%
	F_ArmedF	1 if member of armed forces (ISCO 10), 0 otherwise	2.20%
	F_Skilled	1 if skilled worker (ISCO 5-8), 0 otherwise	49.65%
	F_Unskilled	1 if unskilled elementary worker (ISCO 9), 0 otherwise	22.23%
Mother occupation	M_Top_worker (omitted category)	1 if manager, mid-level professional or supporting worker, 0 otherwise	6.74%
	M_Elem_worker	1 if elementary worker (skilled or unskilled), 0 otherwise	12.54%
	M_Homemaker	1 if homemaker, 0 otherwise	80.73%
Cohort	Pre-war (omitted category)	1 if born 1916–1936, 0 otherwise	17.53%
	War & post-war	1 if born 1937–1958, 0 otherwise	37.03%
	Ec_stabilization	1 if born 1959–1975, 0 otherwise	41.18%
	Democracy	1 if born 1976–1981, 0 otherwise	4.27%

^a^Weights are applied to the descriptive statistics. % in categorical variables, mean and linearized std. error for the continuous ones. ^b^We report the percentages for all the possible values of the variable (in brackets). ^c^For equivalent income and wealth dummies we report mean (and standard deviation in brackets) of income and wealth levels for each quartile

Table 2 displays the estimated coefficients and their standard errors for Eqs. (1)-(3), except those corresponding to interaction terms with cohort and fathers’/mothers’ occupation. In Table 3 we show the adjusted probabilities for interaction terms in Eqs. (1)-(3) and their 95% CI.

**Table 2:** Results of the estimation (without interaction terms). Equations (1)-(3)

Explanatory variables	Eq. (1) Probability of having a higher educational level. Ordered probit estimates	Eq. (2) Probability of having a top occupation. Binary probit estimates	Eq. (3) Probability of reporting good health. Binary probit estimates
Age	-0.039*** (0.002)	-0.005* (0.003)	-0.061*** (0.005)
Female	-0.159*** (0.017)	-0.450*** (0.028)	-1.490*** (0.379)
Father occupation			
F_Supporting	0.169 (0.112)	-0.799*** (0.110)	-2.733*** (0.743)
F_ArmedF	0.308 (0.155)	-0.713*** (0.180)	-2.419*** (0.677)
F_Skilled	-0.790*** (0.102)	-1.343*** (0.103)	-4.494*** (1.188)
F_Unskilled	-1.222*** (0.116)	-1.591*** (0.117)	-5.276*** (1.382)
Mother occupation			
M_Elem_worker	-0.628*** (0.170)	-0.425** (0.175)	-1.483*** (0.413)
M_Homemaker	-0.446*** (0.148)	-0.412*** (0.145)	-1.258*** (0.398)
Cohort			
War & post-war	-0.056 (0.173)	-0.233 (0.181)	-0.825*** (0.275)
Ec_stabilization	-0.270 (0.180)	-0.136 (0.199)	-0.776*** (0.247)
Democracy	-0.577 (0.368)	0.070 (0.412)	0.045 (0.437)
Current economic status			
2^nd^ Quartile Income			0.071* (0.037)
3^rd^ Quartile Income			0.249*** (0.042)
4^th^ Quartile Income			0.375*** (0.056)
2^nd^ Quartile net wealth			0.153*** (0.038)
3^rd^ Quartile net wealth			0.174*** (0.043)
4^th^ Quartile net wealth			0.295*** (0.057)
Residuals			
Equation [1]		0.083*** (0.027)	0.229*** (0.084)
Equation [2]			2.737*** (0.754)
% correct predictions	73.80%	80.96%	76.79%
N° observations	26,832	26,832	26,832

***p < 0.01, **p < 0.05, *p < 0.1

**Table 3:** Adjusted predictions of interaction terms in Equations (1)-(3): probability evaluated at the means

	Pre-war	War & post-war	Ec_stabilization	Democracy
Probability of having a higher educational level (Eq. (1))				
Fathers’occupation				
F_Man&Prof	0.42 (0.35; 0.48)	0.53 (0.49; 0.57)	0.44 (0.41; 0.48)	0.29 (0.13; 0.44)
F_Supporting	0.47 (0.42; 0.53)	0.47 (0.44; 0.51)	0.37 (0.34; 0.40)	0.27 (0.19; 0.35)
F_ArmedF	0.52 (0.43; 0.61)	0.46 (0.39; 0.53)	0.38 (0.31; 0.46)	0.28 (0.10;0.46)
F_Skilled	0.19 (0.15; 0.22)	0.21 (0.19; 0.23)	0.22 (0.21; 0.24)	0.21 (0.16; 0.25)
F_Unskilled	0.10 (0.07; 0.13)	0.12 (0.10; 0.14)	0.13 (0.11; 0.14)	0.09 (0.05; 0.12)
Mothers’occup.				
M_Top_worker	0.37 (0.28; 0.45)	0.29 (0.24; 0.34)	0.28 (0.25; 0.31)	0.27 (0.19; 0.36)
M_Elem_worker	0.20 (0.16; 0.25)	0.24 (0.21; 0.27)	0.23 (0.20; 0.26)	0.15 (0.10; 0.20)
M_Homemaker	0.25 (0.21; 0.28)	0.28 (0.26; 0.29)	0.25 (0.24; 0.27)	0.20 (0.16; 0.24)
Probability of having a top occupation (Eq. (2))				
Fathers’occupation				
F_Man&Prof	0.42 (0.34; 0.50)	0.43 (0.39; 0.48)	0.42 (0.37; 0.47)	0.40 (0.16; 0.64)
F_Supporting	0.17 (0.12; 0.21)	0.24 (0.20; 0.27)	0.18 (0.14; 0.21)	0.13 (0.04; 0.21)
F_ArmedF	0.19 (0.10; 0.28)	0.23 (0.17; 0.30)	0.23 (0.14; 0.32)	0.35 (0.08; 0.79)
F_Skilled	0.07 (0.04; 0.09)	0.10 (0.09; 0.12)	0.10 (0.09; 0.12)	0.08 (0.04; 0.14)
F_Unskilled	0.04 (0.02; 0.06)	0.06 (0.05; 0.08)	0.06 (0.05; 0.08)	0.02 (0.01; 0.04)
Mothers’occup.				
M_Top_worker	0.19 (0.12; 0.26)	0.18 (0.13; 0.22)	0.20 (0.14; 0.25)	0.21 (0.11; 0.32)
M_Elem_worker	0.11 (0.07; 0.14)	0.14 (0.11; 0.17)	0.13 (0.09; 0.16)	0.08 (0.02; 0.17)
M_Homemaker	0.11 (0.09; 0.13)	0.15 (0.14; 0.17)	0.14 (0.12; 0.15)	0.11 (0.06; 0.16)
Probability of reporting a good health status (Eq. (3))				
Fathers’occupation				
F_Man&Prof	0.98 (0.98; 0.99)	0.98 (0.97; 0.99)	0.97 (0.96; 0.98)	--
F_Supporting	0.84 (0.81; 0.86)	0.90 (0.88; 0.92)	0.85 (0.83; 0.87)	--
F_ArmedF	0.87 (0.83; 0.90)	0.90 (0.87; 0.91)	0.89 (0.86; 0.92)	--
F_Skilled	0.51 (0.42; 0.61)	0.68 (0.65; 0.69)	0.68 (0.65; 0.71)	--
F_Unskilled	0.31 (0.14; 0.48)	0.47 (0.35; 0.54)	0.49 (0.39; 0.59)	--
Mothers’occup.				
M_Top_worker	0.91 (0.84; 0.98)	0.87 (0.81; 0.93)	0.92 (0.85; 0.98)	--
M_Elem_worker	0.55 (0.43; 0.67)	0.72 (0.70; 0.76)	0.68 (0.63; 0.74)	--
M_Homemaker	0.62 (0.51; 0.73)	0.80 (0.77; 0.83)	0.78 (0.76; 0.81)	--

95% Confidence Interval in parentheses. (--) Impossible to estimate or not reported because of the small sample size and small variation in the dependent variable

The estimated coefficients for demographic variables (Table 2) are mostly highly significant, and they have the expected sign. Age significantly reduces the probability of having a higher educational level and reporting good or very good health, and it also seems to have a significant –although lower- influence on the probability of being at the top of the occupational ranking. Furthermore, women are less likely than men to reach a high educational attainment, to have top occupations or to report good health.

Parental occupation is very relevant across the three equations, and the effect of father’s occupation exceeds that of mother’s. Compared to those people who have a manager or mid-level professional father, having a father categorized as an skilled or unskilled elementary worker significantly reduces the probability of attaining a higher educational level, having a top occupation or reporting good health. Also, those whose mother is a housewife or an elementary worker tend to report a lower educational level, a lower occupational status and a worse health, compared to people whose mother occupies a managerial position, is a mid-level proffesional or a supporting worker.

Cohort variables do not seem to play a significant role in the estimation of educational and occupational attainments. However, people born during the period 1936–1975 tend to report significantly worse health than those born in the pre-war cohort.

Besides, the current economic situation of the household, which is only included in Eq. (3) –probability of reporting good health-, is highly significant. The higher the quartile of equivalent household income or wealth, the greater the odds of being healthy. Also, non-observable variables represented by $\widehat{u_1}$ and $\widehat{u_2}$ are significant and show the expected sign in Eqs. (2) and (3). Firstly, $\widehat{u_1}$ in Eq. (2) indicates that educational attainments not attributable to parental antecedents positively (and significantly) affect to the probability of having a top occupation. Secondly, $\widehat{u_1}$ and $\widehat{u_2}$ in Eq. (3) indicate that attainments in education and occupation not attributable to parental antecedents influence descendants’ SAH positively and also significantly, as the residuals of previous equations are significant at 99%.

The results shown in Table 3 confirm the large and significant estimated effects of fathers’ occupation on descendants’ education, occupational category and SAH. Descendants of managers and professionals exhibit the highest probabilities of reaching higher education, top occupations and good health across all cohorts. Furthermore, descendants of unskilled worker fathers show lower probabilities of a higher education compared to descendants of skilled workers for all cohorts. This gap is even higher during the democracy period, although the total social gradient was significantly reduced during democracy as probabilities of having a higher educational level for children of managers, professionals, supporting workers and armed forces noticeably decrease for the last cohort. In fact, inequalities rose between the pre-war and the post-war cohort, but they started to decrease just after that point. The legacy of top occupations from father to children is also significant and persistent over time, and differences between children of managers and professionals and children of unskilled elementary workers hardly change after the war except for the democracy cohort, which shows a noticeably increased gap.

Regarding health, the most relevant finding is that the social gap in descendants’ health inherited from the father decreased significantly across cohorts, except perhaps for the youngest cohort, although it is not possible to obtain reliable estimates due to the small sample size in this case. The probability of reporting a good health status for children of unskilled elementary workers increases from 0.31 (95% confidence interval (CI) 0.14–0.48) for the oldest cohort to 0.49 (95% CI 0.39–0.59) for those born during the period 1959–1975. For children of skilled workers, those probabilities increase from 0.51 (95% CI 0.42–0.61) to 0.68 (95% CI 0.65–0.71), respectively. Therefore, children of both skilled and unskilled workers significantly improved their probability of reporting good health across time. Nevertheless, children of managers or mid-level professionals show probabilities ranging from 0.98 (95% CI 0.98–0.99) to 0.97 (95% CI 0.96–0.98). Consequently, discrimination against lowest level workers persists over time.

The influence of the mother on descendants’ outcomes is more limited. The effects on education show that mothers in top occupations had more educated children in the first and the last cohorts, but no significant differences are found in the war and post-war and Stabilization cohorts. It is worth noting that, for all cohorts, children whose mother is a homemaker show a higher probability of attaining a higher educational level compared to those with skilled and unskilled elementary working mothers. However, our models do not find significant effects on descendants’ occupation.

Homemaker mothers also seem to exert some protection effect on their descendants’ health, at least compared to those classified as skilled or unskilled elementary workers. Children of women in top occupations, however, show the highest probabilities of having good health. This gradient remains stable across cohorts, at least until democracy. Again, we have not reported the estimated probabilities for Eq. (3) for the cohort under democracy due to its small sample size (only 442 individuals) and also to the low variability in SAH (only 17 out of the 442 said their health is not good).

## Discussion

Our results clearly indicate that parental occupation (as a measure of parental SES), has a significant impact on the health of children who become adults aged from 28 to 86. These results are consistent with previous studies which focused on elder Spaniards [11, 18, 21]. Once we control for the indirect effects of social circumstances (pathway hypothesis), we prove that there has been a direct impact of early-life conditions on individuals’ health. Besides, this effect is persistent through cohorts and generations.

Fathers and mothers seem to exert different influences, with the influence of the mother’s status on children’s achievements being notoriously lower. The same result was found by Tubeuf and Jusot [11]. The higher the socioeconomic level associated with the father’s occupation, the more likely the individual will be more educated, healthier and occupy a top job, and these effects persist over time. We can also see a social gradient between individuals depending on their mothers’ occupation, but in this case children of homemakers also have an advantage over the children of those mothers at the bottom of the occupational ranking, at least with respect to education and health. This fact could be due to that mother homemakers form a mixed group, which includes women with low qualification but also highly educated women who do not work (because of family obligations or any other reasons). While father’s occupation reflects in most of the cases his educational background, the same is not true for mothers.

Regarding health, the positive influence of mothers as homemakers has been noted in other studies, for instance, to child obesity [27]. This might be due to the value that mothers’ exclusive dedication to the health of their children provides. However, the estimated coefficients in this case might also be reflecting a problem of multicollinearity given that some mothers are homemakers because the husband’s high SES generates enough income to support the family. Nevertheless, as we did not find any significant influence of mothers’ occupation on the probability for children to have a top occupation, we conclude that this is not a serious problem in our case.

Moreover, the inheritage effect for occupational status found in this paper has been previously shown for other countries [28, 29] and also for Spain [30], and indicate that occupational stratification flows from one generation to the next. Also, we have found a positive and significant influence of individuals’ economic situation on their self-reported health, similarly to what other studies proved for several European countries [31].

Social inequalities in educational attainment linked to fathers’ SES between the top and the bottom of the social scale seem to have grown between the pre-war and the post-war cohorts, but they started to decrease from the Stabilization Plan cohort. However, differences in educational attainment between descendants of unskilled and skilled workers not only are visible across time, but they seem to have widened under democracy. This is an unexpected result, as education grants were widely promoted over this period. Lastly, the health gap between the children of unskilled and skilled workers has significantly decreased since the Stabilization Plan cohort. This reduction in health inequalities could possibly be related, at least for the third cohort, to the reform of the social security system covering health care for workers and their families (Ley de Bases de la Seguridad Social, 1963). Our results suggest that health inequalities due to the legacy effect have declined more than inherited educational and occupational inequalities over time.

An advantage of this study is its large sample and unusually detailed information on household income and wealth. Since the design of the survey gave more weight to high SES households, we were better able than previous studies to track the legacy of the most privileged parents to their offspring. However, there are some limitations linked to the dataset. The question on parental occupation in the survey refers to the job held during a parent’s active life, not during the respondent’s childhood. Fortunately for our purposes, labour mobility in Spain traditionally has been extremely limited, so we can assume that the occupation reported in the survey is a decent proxy. We also lack information on parental education and income; thus we are unable to incorporate the educational persistence hypothesis in our first equation, despite the fact that Spain is known to be one of the OECD countries where this persistence is most pronounced [32]. Lastly, neither could we incorporate parental health status into our model. Another possible bias is selection by mortality. We only observe the survivors, who may have been less affected by poor inheritance. This would be a downward bias, therefore, and the real effects of parental legacy might be even greater than those we have estimated. Moreover, Eqs. (1) and (2) do not take into account that individuals’ health may affect educational and job attainment, as the reverse causality hypothesis points out. The SAH equation also omits relevant variables not collected in the survey, along with other unobservable heterogeneity. The connections between education and health are not so simple in real life as in the model [33]. Furthermore, it may be that not only occupation but also job security affects health, although this phenomenon does not seem as important in Spain as elsewhere [34, 35].

The selection of the sample may also be seen as a limitation of the study. However, it will hardly affect our results, since the proportion of people under 28 years is quite low (1.77% of the sample). Furthermore, people aged over 86 account for only 0.81% of the sample and many of them are living in their descendants’ homes. In the EFF, more than a half of the sample of people aged 87 or older are neither the reference person nor his/her partner.

The confusion of the age effect with the cohort effect is also a potential limitation. Individuals in our sample who were born in the post-war period are aged from 45 to 72, and the pre-war cohort is older than 66. Most of the individuals belonging to cohorts under democracy (28–33 years old) are younger at the time of the survey than cohorts of the Stabilization Plan period (28–51 years old), as the timespan of the survey is only 8 years. Thus, there are no persons in the sample born under democracy older than 33, and therefore we cannot compare older persons born under Franco’s dictatorship and older persons born under democracy. Age at the time of the survey only overlaps for adjacent cohorts. We have not reported the estimated probabilities for Eq. (3) for the cohort under democracy because the sample size is small and the variability in SAH is very low. Future research will be able to follow these cohorts as they grow older, since latent health problems may surface later in life due to poor living conditions in childhood. Other than that, it seems clear that the children of the socially privileged, at least until the democracy cohorts, maintain privileges in health during adulthood.

## Conclusions

By using a large data sample, which includes unusually detailed information on household income and wealth and covers cohorts of adults born in different decades, we conclude that parental occupation has a significant direct impact on individuals’ health. Socioeconomic status is transmitted from one generation to the next through different paths, but adult’s health is, to a certain extent, a consequence of the family socioeconomic position during childhood.

Although health inequalities in adult Spaniards are partly due to socioeconomic circumstances in childhood, great progress has been made during the twentieth century, so that the impact of parental SES on individuals’ health has been significantly tempered for those at the bottom of the social scale. Nevertheless, since discrimination against unskilled workers’ descendants persists over time, more efforts focused on the improvement of living conditions for most socioeconomically disadvantaged are needed in order to further reduce social inequalities in health. This is an important message for social policy makers: actual social policies may have persistent effects and are potentially useful to promote health of generations ahead.

## Abbreviations

CI: Confidence interval
EFF: Spanish Survey of Household Finances (Encuesta Financiera de las Familias)
ISCO: International Standard Classification of Occupations
OECD: Organization of Economic Cooperation and Development
SAH: Self-assessed health
SES: Socioeconomic status
SHARE: Survey of Health, Aging and Retirement in Europe
